# Profiling of ob/ob mice skeletal muscle exosome-like vesicles demonstrates combined action of miRNAs, proteins and lipids to modulate lipid homeostasis in recipient cells

**DOI:** 10.1038/s41598-021-00983-3

**Published:** 2021-11-03

**Authors:** Audrey Jalabert, Laura Reininger, Emmanuelle Berger, Yohann Coute, Emmanuelle Meugnier, Alexis Forterre, Elizabeth Errazuriz-Cerda, Alain Geloen, Myriam Aouadi, Karim Bouzakri, Jennifer Rieusset, Sophie Rome

**Affiliations:** 1 CarMeN Laboratory (INSERM 1060, INRAE 1397, INSA), Lyon-Sud Faculty of Medicine, University of Lyon, Oullins, France; 2 UMR DIATHEC, EA 7294, Centre Européen d’Etude du Diabète, Université de Strasbourg, Strasbourg, France; 3 UMR Ecologie Microbienne Lyon (LEM), CNRS 5557, INRAE 1418, University of Lyon, VetAgro Sup, Villeurbanne, France; 4 Univ. Grenoble-Alpes, Inserm, CEA, UMR BioSanté U1292, CNRS CEA FR2048, Grenoble, France; 5 CIQLE, Claude Bernard Lyon 1 University, Lyon, France; 6 Centre for Infectious Medicine, Department of Medicine, Karolinska Institutet, Huddinge, Sweden; 7 Institut of Functional Genomic, ENS-Lyon, University of Lyon, CNRS 5242, INRAE Lyon, France

**Keywords:** Biochemistry, Molecular medicine

## Abstract

We have determined the lipid, protein and miRNA composition of skeletal muscle (SkM)-released extracellular vesicles (ELVs) from Ob/ob (OB) *vs* wild-type (WT) mice. The results showed that atrophic insulin-resistant OB-SkM released less ELVs than WT-SkM, highlighted by a RAB35 decrease and an increase in intramuscular cholesterol content. Proteomic analyses of OB-ELVs revealed a group of 37 proteins functionally connected, involved in lipid oxidation and with catalytic activities. OB-ELVs had modified contents for phosphatidylcholine (PC 34-4, PC 40-3 and PC 34-0), sphingomyelin (Sm d18:1/18:1) and ceramides (Cer d18:1/18:0) and were enriched in cholesterol, likely to alleviated intracellular accumulation. Surprisingly many ELV miRNAs had a nuclear addressing sequence, and targeted genes encoding proteins with nuclear activities. Interestingly, SkM-ELV miRNA did not target mitochondria. The most significant function targeted by the 7 miRNAs altered in OB-ELVs was lipid metabolism. In agreement, OB-ELVs induced lipid storage in recipient adipocytes and increased lipid up-take and fatty acid oxidation in recipient muscle cells. In addition, OB-ELVs altered insulin-sensitivity and induced atrophy in muscle cells, reproducing the phenotype of the releasing OB muscles. These data suggest for the first time, a cross-talk between muscle cells and adipocytes, through the SkM-ELV route, in favor of adipose tissue expansion.

## Introduction

Exosome-like vesicles ELVs represent a discrete population of 50–120 nm-sized vesicles formed during endosomal maturation. Inward buddings of the late endosomal limiting membranes form the multivesicular bodies (MVBs) which contain intraluminal vesicles (i.e.; ILVs). MVBs can fuse either with lysosomes for ILV content degradation or with the plasma membrane to release ILVs in the extracellular milieu which are then named ELVs. MVB destiny is strongly connected to cell metabolic status i.e.; starvation induces intracellular nutrient recycling and in that context MVBs preferentially fuse with amphisome/lysosome^1^ and the release of ELVs is decreased^2^. On the other hand, unwanted and damaged materials are released into the extracellular environment through MVB fusion with the plasma membrane during high metabolic turn-over such as proliferation/differentiation. Preventing ELV release is deleterious and is a new therapeutic strategy to treat cancer^3^. ELVs contain endosomal lipids, proteins targeted for degradation (e.g.; ubiquitination, sumoylation), signaling proteins^4^ including the insulin-sensitive AKT protein^5^, and various RNA species^6–8^. Interestingly, it has been demonstrated that ELVs can horizontally transfer their cargoes into other cell types and therefore can regulate their fate^7^. With the numerous proteins, lipids, and nucleic acids they carry, ELVs can affect multiple cellular pathways in target cells and they represent a potential mode of intercellular communication which acts in synergy with soluble growth factors and hormones. Previous studies have demonstrated that skeletal muscle releases ELVs (SkM-ELVs), in addition to myokines, and have suggested that SkM-ELVs would act as deleterious signals during the development of insulin-resistance (IR) associated to obesity-induced diabetes^9^. Indeed, it was demonstrated that SkM insulin-resistance induced by a diet enriched in palm oil triggered the release of a new population of SkM-ELVs enriched in palmitate, able to spread the deleterious effect of this lipid between muscle cells^10^. The same population of SkM-ELVs could also transfer specific miRNAs into recipient pancreatic beta cells to induce their proliferation, suggesting that muscle-released ELVs might contribute to adaptations in beta cell mass occurring during IR development associated with obesity in mice^11^. Beside these studies which are based on the consumption of a very specific diet (i.e.; enriched in palm oil^10^) neither the consequences of obesity on the release and composition of SkM-ELVs, nor whether obesity affected their biological properties have been evaluated. Indeed, for other cell types like the adipocytes, it was demonstrated that obesity affected adipocyte-released ELV content which exported a high proportion of obesity and IR-related proteins/lipids/RNAs from adipocytes able to modify the metabolic responses of other insulin-sensitive target cells^12^. Therefore, as obesity affects lipid and glucose metabolism also in skeletal muscle^13^ we postulated that SkM-ELV composition and properties would be also modified and might be important mediators of obesity-associated metabolic complications such as insulin-resistance. In order to challenge this hypothesis, we have considered the leptin-deficient (*ob/ob*) mice as a model of obesity not related to the diet composition, considering for the first time in the same study SkM-ELV lipids, proteins and miRNAs. Then, we have determined the biological processes affected by ELVs released from ob/ob muscles, in muscle cells and adipocytes.

## Material and methods

### Ob/ob mice

Male leptin-deficient (*ob/ob*) mice on a C57BL/6 background and C57BL/6 control mice were purchased from Harlan at 4 weeks of age, 22 °C and with a 12-h light/dark cycle. Animal procedures were conducted in accordance with the institutional guidelines for the care and use of laboratory animals and was carried out in compliance with the ARRIVE guidelines (https://www.google.com/search?client=firefox-b-d&q=pubmed). This study was approved by the ethics committee of AniRA-PBES core facility at ENS-Lyon, France. Mice were fed for 12 weeks with a standard chow diet (SD, 57% carbohydrate, 5% fat and 18% protein; Harlan). At the end of the protocols, blood was withdrawn at fed state, then, animals were killed by cervical dislocation, and quadricep (Quad) and gastrocnemus (Gast) muscles were rapidly excised. Given the low concentration of SkM-ELVs, it was not possible to use the same muscle for all animals and we have chosen to consider both Gast of Quad for SkM-ELV purification, which are two muscles almost exclusively composed of type II fibers and have the same metabolic alterations during the development of obesity.

### Protein quantification by western blot

Gast were frozen in liquid nitrogen and proteins were extracted in RIPA lysis buffer [PBS, 0.1% SDS (Promega), 0.5% Sodium Deoxycholate (Sigma-Aldrich),1% Nonidet NP40 (Sigma-Aldrich), 5 mM EDTA (Sigma-Aldrich), 1 mM Na3VO4 (Sigma-Aldrich), 20 mM NaF (Sigma-Aldrich), 1 mM DTT (Sigma-Aldrich), cocktails of Protease inhibitors (Sigma-Aldrich)]. Proteins were denatured and loaded in a 10% SDS-PAGE gels (10 μg) and further transferred onto nitrocellulose PVDF membranes. Membranes were incubated with antibodies overnight (Table S1) then washed in TBS-Tween 3‰ and further incubated with anti-Rabbit Horseradish Peroxidase conjugated secondary antibody (#172-1019, Bio-Rad). Signals were revealed with Immuno detection kit ECL Luminata Classico (Millipore) and the imager Molecular Image® ChemiDoc™XRS + (Bio-Rad). Proteins quantification was performed by using ImageLab 3.0 (Bio-Rad).

### Exosome-like vesicle released from skeletal muscle explants

Quad or Gast from *ob/ob* and control mice (n = 5) were cut into small pieces to remove all contaminant tissues and incubated for 24 h in serum-free DMEM at 37 °C under 5% CO2 atmosphere. Conditioned medium was centrifuged at 300*g* for 20 min and 2000*g* for 20 min to remove dead cells and the supernatent was further centrifugated at 10,000*g* for 30 min to remove large particles and organelles. The resulting supernatant was filtered through a 0.22 µm filter. Exosome-like vesicles (ELV) were pelleted by ultracentrifugation at 100,000*g* for 70 min at + 4 °C (Beckman-Coulter, Optima^tm^ L-80-XP ultracentrifugy, type 50-2Ti rotor). The pellets from a single sample were pooled, resuspended in 25 ml PBS and again centrifuged at 100,000×*g* for 70 min. The resulting pellet containing the SkM-ELVs was finally resuspended in 50 µl PBS. SkM-ELV protein content was quantified by using a Bradford protein assay. SkM-ELV size distributions were determined by NanoTracking Analyses which allows the determination of a size distribution profile of small particles with a diameter of approximately 50–500 nm (nm) in liquid suspension (Malvern Panalytical).

### Transmission electron microscopy (TEM)

SkM-ELV were observed by TEM as previously descrived^38^. SkM-ELVs in PBS were adsorbed on 200 Mesh nickel grids coated with formar-C. Immunogold labelling was performed by flotation of grids on drops of reactive media. Non-specific sites were coated with 1% BSA in 50 mM Tris–HCL, pH 7.4 for 10 min at RT. Antibody incubation was carried out for 4 h at 4 °C in a wet chamber with mouse monoclonal antibody raised against CD81 (sc-166028, Santa Cruz Biotechnology) (dilution 1/50) in 1%BSA, 50 mM Tris–HCL, pH 7.4. Grids were successively washed once in 50 mM Tris–HCL, pH 7.4 and pH 8.2 at RT. They were then preincubated with 1% BSA in 50 mM Tris–HCL, pH 8.2 for 10 min at RT and labelled with a goat anti mouse IgG gold-conjugated 10 nm, (Tebu bio, France) diluted 1/80 in 1% BSA-, 50 mM Tris–HCL, pH 8.2 in a wet chamber for 45 min. Grids were successively washed once in 50 mM Tris–HCL, pH 8.2 then pH 7.4 and in filtrated distilled water at RT. Finally, grids with suspensions were colored with 2% phosphotunstic acid for 2 min and examined using a JEM Jeol 1400 transmission electron microscope (Tokyo, Japan) equipped with a Orius 600 camera (USA).

SkM from lean and OB mice were fixed in 2% glutaraldehyde, washed 3 times at 4 °C (saccharose 0.4 M/0.2 M Na C-HCl-Cacodylate-HCl, pH = 7.4) and post-fixed at 4 °C (2% OsO4/0.3 M Na C-HCl Cacodylate-HCl pH = 7.4). Then muscles were dehydrated with an increasing ethanol gradient (5 min in 30%, 50%, 70%, 95% ,and 3 times for 10 min in absolute ethanol). Impregnation was performed with Epon A (50%), Epon B (50%), DMP30 (1,7%). Inclusion was obtained by polymerization at 60 °C for 72 h. Ultrathin Sects. (70 nm) were cut using a UC7 (Leica) ultra-microtome, mounted on 200 mesh copper grids coated with 1°/°° polylysine, and stabilized for 1 day at room temperature, and, contrasted with uranyl acetate and lead citrate. Sections were examinated with a Jeol 1400JEM (Tokyo, Japan) transmission electron microscope equipped with a Orius 600 camera and Digital Micrograph.

### Lipid identification and quantification

Total lipids were extracted from Gast and from SkM-ELVs released from the same Gast muscle^14^. Individual phospholipid classes and their molecular species were quantified by ESI–MS/MS (API3000, TQ, Applied Biosystems-Sciex, Concord, Ontario, Canada)^15^. Cholesterol analysis was adapted from^16^ and performed on a mass spectrometer (Agilent 5975 inert XL) in series with the gas chromatography set up for detection of positive ions.

### Extraction of miRNAs from quadriceps explants and ELVs

We used 100 µg of protein-containing SkM-ELVs for total RNA extraction. Total RNA was extracted with TriPure Isolation Reagent (Roche Applied Science, France). RNA was quantified with a NanoDrop spectrophotometer (Labtech, France). We obtained 186.6 ± 7.18 ng of total RNA. Frozen Quad were crushed in liquid nitrogen and total RNA was extracted as in^17^.

### Identification and quantification of mature miRNAs

Expression of mature miRNAs was measured by using the TaqMan® Low Density Arrays V2 containing 590 small RNAs with Applied Biosystems 7900HT Fast Real-time PCR system, as previously described in^38^. For SkM-ELV miRNA profiling, 33 ng of total RNA were used for multiplex reverse transcription (RT). Pre-amplification was realized with 2.5 µl of cDNA using the TaqMan Megaplex Pre-Amp system (Applied Biosystems) according to manufacturer’s instructions. For Quad miRNA profiling, 900 ng were used for multiplex RT and the resulting cDNA was not pre-amplified. Then each cDNA reaction was mixed with TaqMan Universal PCR Master mix and loaded into the corresponding fill port. Individual singleplex PCR reactions were performed in 384-well reaction plates with Applied Biosystems 7900HT Fast Real-time PCR system. The level of miRNA was measured using Ct (threshold cycle) determined by RQ Manager. For each miRNA, the threshold cycle (Ct) was calculated by the ABI 7900 Sequence Detection System software. We used the mean expression level of all fully detected miRNAs for normalization. Comparison between groups were made by using the student *t*-test (p < 0.05) on normalized data, to select the differentially expressed miRNAs between *ob/ob* mice and wild type. Data are expressed in Ct values, which are inversely proportional to miRNA concentrations.

### Gene expression quantification by qRT-PCR

qReal-time PCR was performed using ABsolute QPCR SYBR Green ROX Mix (Abgene, Courtaboeuf, France) with a Rotor-Gene 6000 system (Corbett Life Science, Paris, France). Data are expressed as mean ± SEM. Comparisons were analysed using Student’s *t*-test. Significance was defined as *p* value of < 0.05. The list of PCR primers is given in Table S1.

### Quantitative mass spectrometry-based proteomic analyses of ELVs released from mouse quadriceps

SkM-ELV proteins were resuspended in Laemmli buffer, stacked in the top of a SDS-PAGE gel (NuPAGE 4–12%, ThermoFisher Scientific) and stained with Coomassie blue (R250, Bio-Rad) before being in-gel digested using modified trypsin (Promega, sequencing grade) as previously described^18^. Resulting peptides were analyzed by online nanoLC-MS/MS (UltiMate 3000 and LTQ-Orbitrap Velos Pro). For this, peptides were sampled on a 300 µm × 5 mm PepMap C18 precolumn (Thermo Scientific) and separated on 75 µm × 150 mm and 75 µm × 250 mm C18 columns (Gemini C18, 3 µm, Phenomenex) using a 120-min gradient. Two analytical replicates for each biological replicate (n = 3) were performed. MS and MS/MS data were acquired using Xcalibur (Thermo Scientific). Mascot Distiller (Matrix Science) was used to produce mgf files before identification of peptides and proteins using Mascot (version 2.7) through concomitant searches against Uniprot (*Mus musculus* taxonomy, September 2019 version), classical contaminants database (homemade) and the corresponding reversed databases. The Proline software^19^ was used to filter the results (conservation of rank 1 peptides, peptide length ≥ 6 amino acids, peptide-spectrum match identification FDR < 1% as calculated on peptide scores by employing the reverse database strategy, minimum peptide-spectrum-match score of 25, and minimum of 1 specific peptide per identified protein group). Proline was then used to perform a compilation, grouping and MS1 quantification of the identified protein groups. Statistical analyses were performed using ProStaR^20^. Proteins identified in the reverse and contaminant databases, proteins identified with only one peptide and proteins exhibiting less than 6 abundance values in one condition were discarded from the list. After log_2_ transformation, abundance values were normalized by median centering before missing value imputation (slsa algorithm for partially observed values in the condition). Statistical testing was conducted using limma test. Differentially-expressed proteins were sorted out using a log_2_ (fold change) cut-off of 1 and a *p*-value cut-off of 0.01, allowing to reach a FDR inferior to 2% according to the Benjamini–Hochberg procedure. Only proteins identified in 3 biological replicates with a minimum of 2 spectral counts (SC) in 1 biological replicate were considered. For quantitative comparison of WT-ELV and OB-ELV proteomes, we used a beta-binomial test specifically developed to test the significance of differential protein abundances expressed in SC.

### Effect of SkM- ELVs on 3T3-L1 cell proliferation and lipogenesis

To determine the effect of SkM-ELVs on 3T3 cell proliferative and lipogenesis capacities, we used the xCELLigence live cell analysis System (Roche Applied Science) which offers dynamic live cell monitoring, as previously described in^30^. The System measures electrical impedance across interdigitated micro-electrodes integrated on the bottom of tissue culture E-Plates. One day after plating, cells were grown in DMEM 4.5 g/l glucose supplemented with 2.5% ELV-depleted FBS, and incubated with 2 µg SkM-ELVs. The impedance was measured every 15 min for 48 h. Impedance was represented by the cell index (CI) values ((Zi-Z0) [Ohm]/15[Ohm]; Z0: background resistance, Zi: individual time point resistance) and the normalized cell index was calculated as the cell index CIti at a given time point divided by the cell index CInml-time at the normalization time point (nml_time). At the end of the experiment, cells were trypsinized and total RNA were extracted for mRNA level quantification by qRT-PCR.

### Bioinformatics analysis of miRNA target genes and protein functions

Heatmap showing the significantly enriched miRNAs in SkM-ELVs and muscle from OB *vs* mice were created by using ClustVis (https://biit.cs.ut.ee/clustvis/). miRNA target genes were predicted by using TargetScan 6.1 (http://www.targetscan.org/). We have focused this study on miRNA binding sites conserved among species in order to reduce the number of false positive target genes. PANTHER (http://pantherdb.org) was used to analyze the set of proteins contained in SkM-ELVs (G.O functions, cellular pathways, and reactome), and clustering was performed with MORPHEUS (https://software.broadinstitute.org/morpheus/).

### Stastitical analyses

Unpaired two-tailed Student's *t*-tests was used to compare the means of 2 independent samples. Data are expressed as mean ± SEM. The cutoff for significance level was set at *p* < 0.05. Correlations analyses were performed using R (V3.5) using a non-parametric approach (Kendall rank correlation coefficient).

## Results

### OB mice muscle atrophy and insulin-resistance are associated with a decrease in ELV release

**Figure 1:**
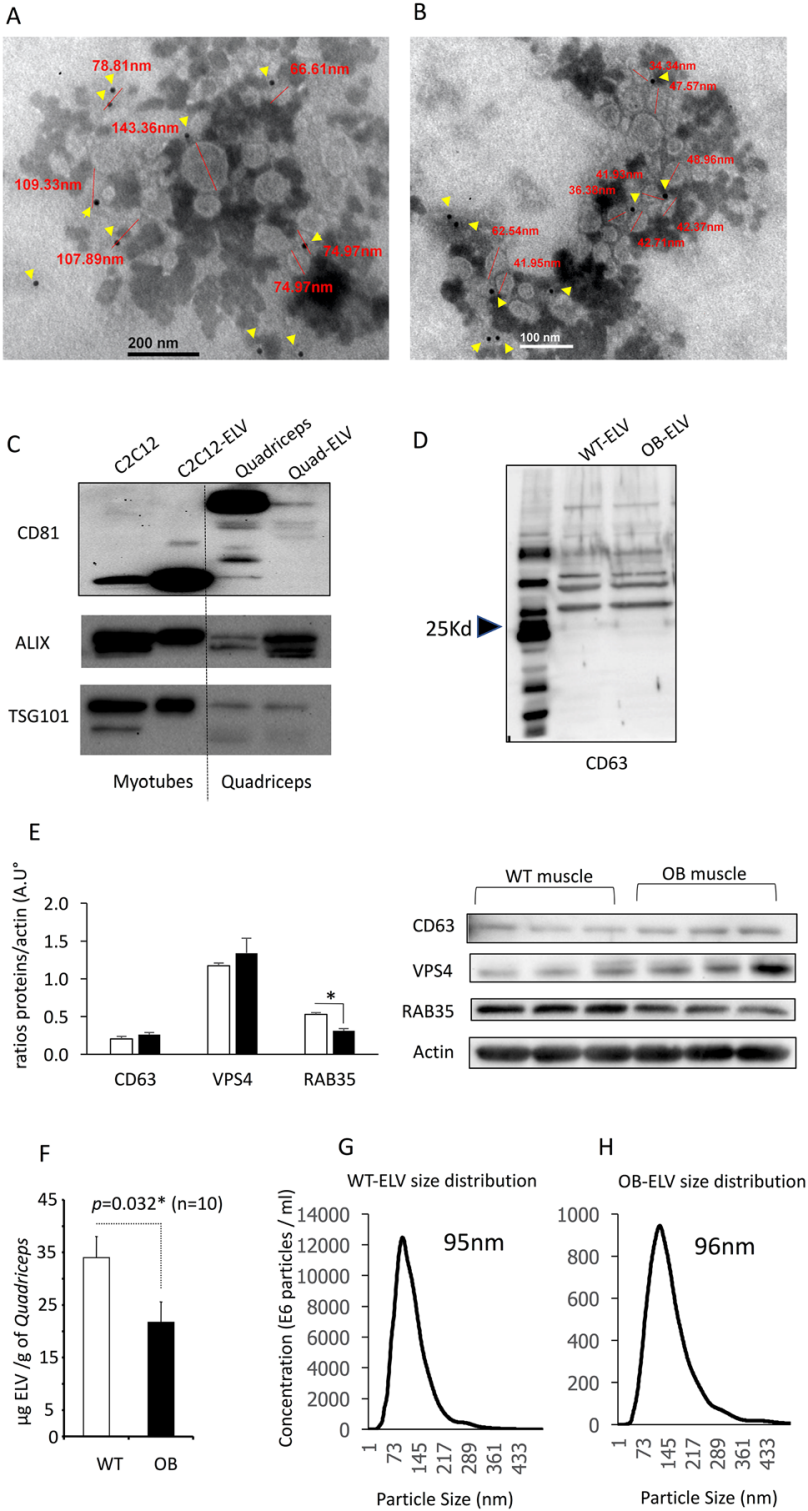
Characterization of SkM-derived exosome-like vesicles (ELVs). (**A**,**B**) Transmission electron microscopy showing WT-ELVs (**A**) and OB-ELVs (**B**) detected with anti-CD81 (yellow arrows = CD81-gold particles (15 nm), the sizes of labelled ELVs are indicated in red). (**C**) WB to identify SkM-ELVs specific proteins (CD81, TSG101, ALIX) in C2C12 and C2C12-released-ELVs, and in WT-Quad and WT-Quad-released ELVs. The same quantity of proteins was loaded. (**D**) WB to show that OB-ELVs and WT-ELVs expressed the exosomal marker CD63. (**E**) WB showing the levels of proteins involved in SkM-ELV release, in WT-Quad vs OB-Quad. (**F**) Quantification of ELVs released from WT-Quad or OB-Quad estimated from their quantity of proteins (Bradford assay). (**G**,**H**) SkM-ELVs size distribution quantified by Nano-tracking analyses. (* = *p* < 0.05 (student t-test)). WB originals are in Fig. S2.

At 12 weeks of age, *ob/ob* (OB) mice presented a marked obesity (Fig. S1A), hyperglycemia (Fig. S1B), liver steatosis (Fig. S1C), and altered insulin-induced AKT phosphorylation in Gast, compared to wild type (WT) littermates (Fig. S1D). Leptin deficiency was associated with a strong reduction of both Quad and Gast weights (Fig. S1E, Fig. S1F). TEM images showed that SkM-ELVs released from muscle explants were CD81 positive (Fig. 1A,B) and contained TSG101 and ALIX proteins (Fig. 1C). They also expressed CD63 at their surface confirming the endosomal origin of the SkM-ELV pellet (Fig. 1D). In muscle, the levels of proteins involved exosome biogenesis (VPS4, CD63) were not different whereas the RAB35 protein, previously demonstrated as a major actor of ELV release, was decreased^21^ (Fig. 1E). In agreement, OB-Quad released significantly less SkM-ELVs *vs* WT-Quad, determined as μg equivalent proteins (Fig. 1F) or by measuring the total quantity of WT- and OB-ELVs by NTA (9.83 10^11^ *vs* 5.70 10^10^, respectively ), without modification of ELV sizes (Fig. 1G,H). Therefore, these data indicated that in OB mice, obesity does not affect ELV biogenesis but affects their release.

### OB muscle ELV are enriched in proteins involved in lipid metabolism

**Figure 2:**
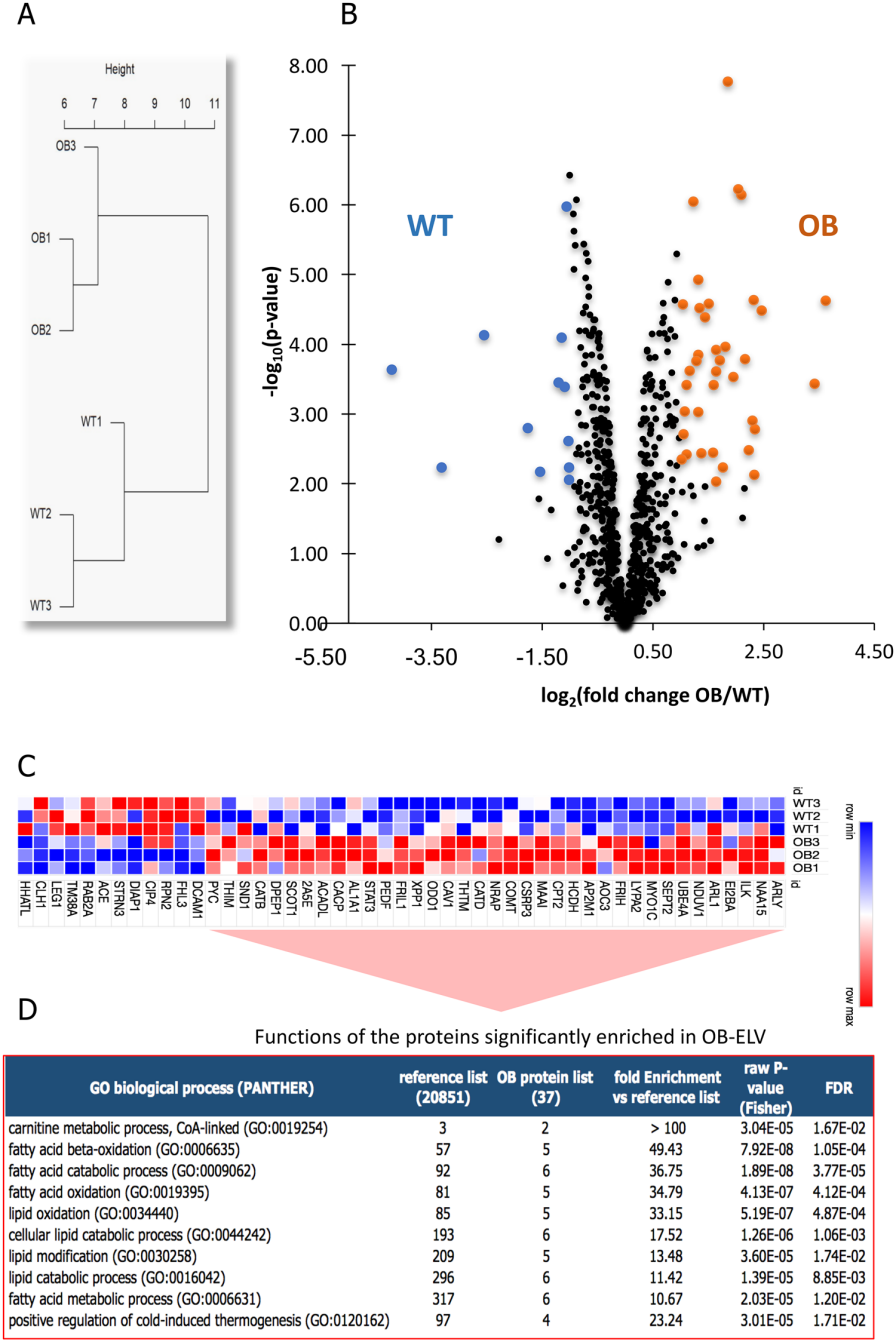
Impact of obesity on SkM-ELV protein profile. The full protein datasets are in Table S2. (**A**) Pie chart showing the intracellular localizations of the 798 SkM-ELV proteins. Only the significant pathways with more than 150 genes are shown. (**B**) Pie chart showing the significant GO metabolic pathways controlled by the 798 SkM-ELV proteins. Only the main significant metabolic pathways with more than 15 genes in the pathway are shown. Redundant pathways have been removed. (**C**) Clustering of the 798 SkM-ELV proteins, based on their abundance, in OB- and WT-ELVs. (**D**) Volcano plot showing statistical significance (*p*-values) versus magnitude of changes (fold changes OB/WT expressed in log2). Red and blue dots represent proteins found more abundant in OB-ELVs and WT-ELVs, respectively (*p*-value ≤ 0.01 and fold change ≥ 2). (**E**) Clustering focused on the subset of proteins differentially abundant in OB-ELVs *vs* WT-ELVs. (**F**) Significant Biological functions associated with the 37 proteins enriched in OB-ELVs *vs* WT-ELVs.

SkM-ELVs released from quadriceps of OB and WT mice were compared using mass spectrometry-based label-free quantitative proteomics. This analysis allowed to map 798 proteins identified with at least two peptides (Table S2). Functional enrichment analyses were performed to determine their molecular functions, their localizations and their biological activities. Data indicated that SkM-ELV proteins were mainly located in intracellular organelles/vesicles/exosomes/endosomes, confirming their endosomal origin (Fig. 2A). In addition, 166 were proteins located in mitochondria, and 377 proteins in the nucleus or had perinuclear localization (Fig. 2A). Analyses of their molecular functions showed that 453 SkM-ELV proteins had catalytic activities and that 59 were kinases, 39 were transferases, 32 were dehydrogenases, and 30 were ligases (Table S2). Beside their involvement in muscle maintenance/development, half of them were involved in metabolic processes, mainly protein, nitrogen compound and lipid metabolisms (Fig. 2B, Table S2).

Hierarchical clustering based on the abundance of proteins showed a clear separation between OB-ELVs and WT-ELVs (Fig. 2C) demonstrating that obesity altered the protein content of SkM-ELVs. Forty-nine proteins were found differentially abundant depending on whether SkM-ELVs originated from OB or WT mice (Fig. 2D,E, Table S2). Protein–protein enrichment *p*-value calculated by using STRING software was 0.016537 for the 37 enriched proteins in OB-ELVs, demonstrating that they were functionally connected as a group. The 37 proteins were significantly enriched in proteins involved in lipid oxidation (Fig. 2F) and 29 of them had catalytic activities (raw *p* value = 3.00E−10, FDR = 1.42E−06). On the contrary, there were no significant interactions among the 12 proteins enriched in WT-ELV *vs* OB-ELVs, and these proteins were not associated with a significant cellular function.

### SkM-ELVs from OB skeletal muscle have altered lipid profiles

**Figure 3:**
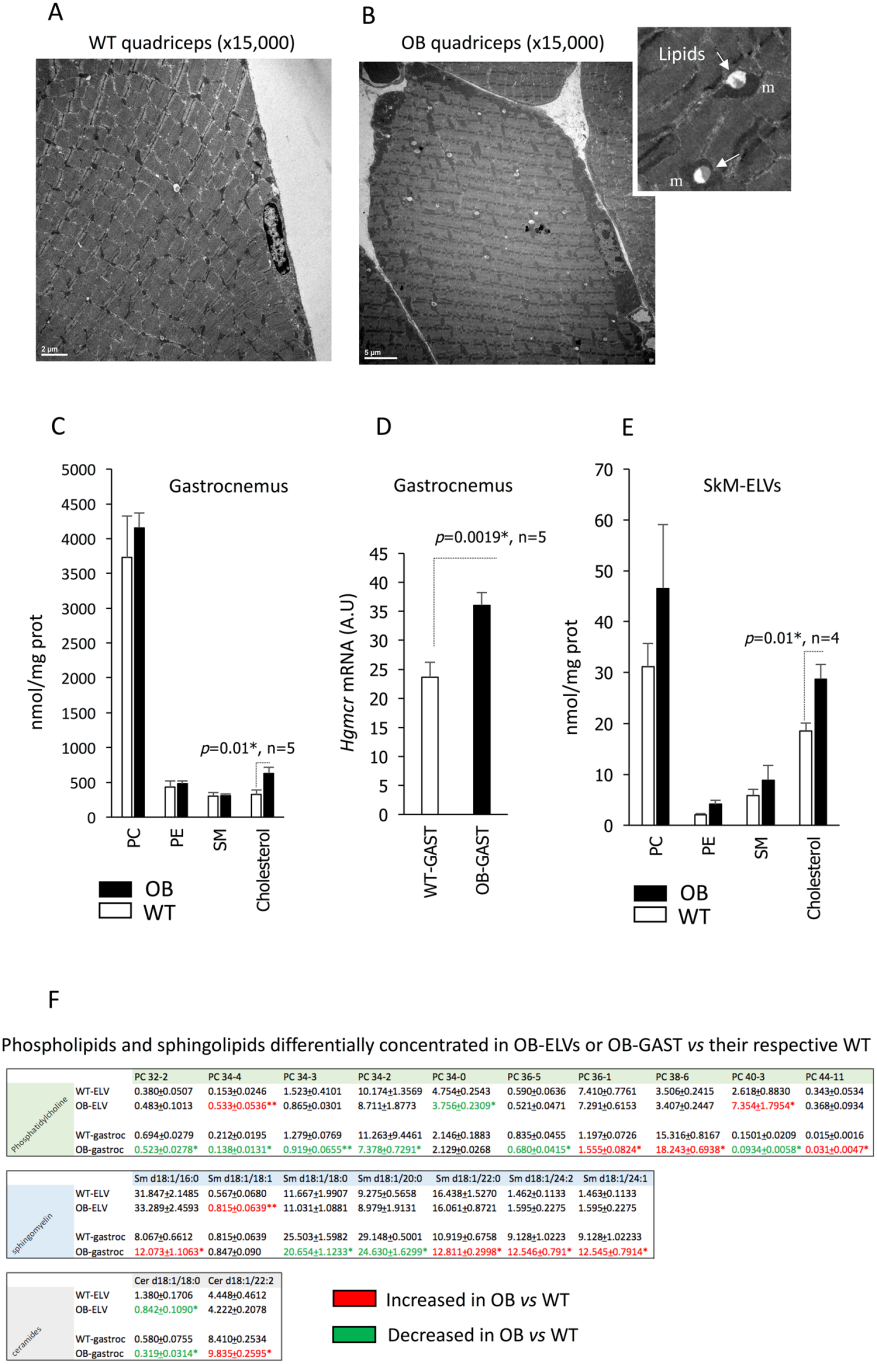
Impact of obesity on SkM-ELV lipid profile. (**A**,**B**) TEM images of Quad muscle from WT (**A**) or OB (**B**) mice. (**C**) Quantification of phospholipids, sphingolipids and cholesterol in Gast from WT and OB mice. (**D**) Quantification of *Hmgcr* mRNA in WT- and OB-Gast (* = p < 0.05, student t-test). (**E**) Quantification of phospholipids, sphingolipids and cholesterol in WT-ELVs and OB-ELVs released from Gast. (**F**) Lipids differentially concentrated in OB-ELVs or OB-GAST *vs* their respective WT. Data are expressed as % of total PC, or total SM or total Ceramides identified and quantified from the same concentration of starting material. Significant distributions are determined from student *t*-test (* = p < 0.05, ** = p < 0.01). red: increased *vs* control; green: decreased *vs* control.

As OB-Gast showed increased lipid deposition compared to WT-Gast (Fig. 3A,B) demonstrating that the lipid metabolism was affected in OB mice, we wondered whether the levels of lipids involved in SkM-ELV biogenesis would be also affected. As showed on Fig. 3C, OB-Gast had higher level of cholesterol than WT-Gast. This result correlated with the increase in 3-hydroxy-3-methylglutaryl-CoA reductase (HMGCR) mRNA in OB muscle (Fig. 3D), the rate-limiting enzyme for cholesterol synthesis. On the contrary, the total levels of phospholipids (phosphatidylcholine (PC), phosphatidylethanolamine (PE)) and sphingomyelin (SM) were not affected in OB-Gast (Fig. 3C). SkM-ELVs were enriched in sphingomyelin (SM) and cholesterol, compared to muscle cells, 2 lipids involved in the formation and release of ELVs^22^ (Fig. 3E). OB-ELVs had increased content of cholesterol compared with WT-ELVs (Fig. 3E) but no modification in PC, PE and SM content. However, the level of specific subspecies of ceramides, SM and PC were modified in OB-Gast *vs* WT-Gast (Fig. 3F). Interestingly, these variations were not always reflected in the lipid composition of OB-ELVs which were less affected by obesity than OB-Gast (Fig. 3F). OB-ELVs had modified content for specific Phosphatidylcholine (PC 34-4, PC 40-3 (enriched) and PC 34-0 (depleted)), sphingomyelin (Sm d18:1/18:1 (enriched)) and ceramides (Cer d18:1/18:0 (depleted)). These data demonstrated for the first time a strong selection for lipid export in SkM-ELVs, which is altered in obese SkM.

### Obesity altered the concentration of specific miRNAs in OB-ELVs

**Figure 4:**
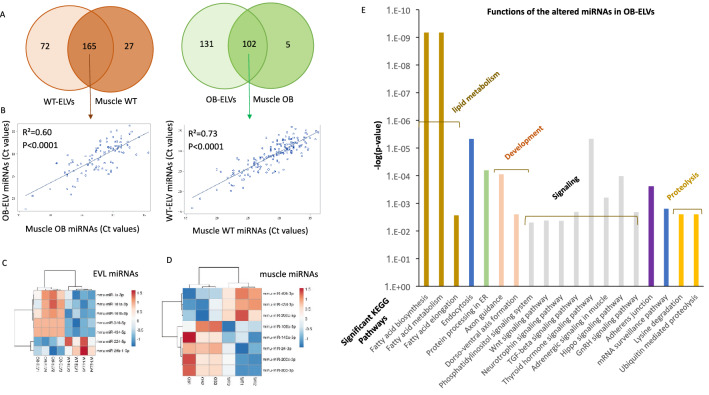
Altered miRNA content in OB-ELVs vs WT-ELV. (**A**) Venn Diagram showing the number of miRNAs commonly expressed between muscle and SkM-ELVs, or specific to each compartment. Given the low number of animals (n = 4) we have considered only the miRNAs expressed in all mice for each diagram. The mean Ct values of all miRNAs expressed in WT or OB muscle was not significantly different. (**B**) Correlations between the Ct values of miRNAs commonly identified between SkM-ELVs and Quad. (**C**) Heatmap showing the differentially concentrated miRNAs in SkM-ELVs or muscle (**D**), between OB and WT mice. (**E**) The significant KEGG pathways containing target genes from the 7 OB-ELV miRNAs (in **C**) were determined by using DIANA miRPath. The 20 most significant KEGG pathways in terms of lowest *p*-values are shown.

**Figure 5:**
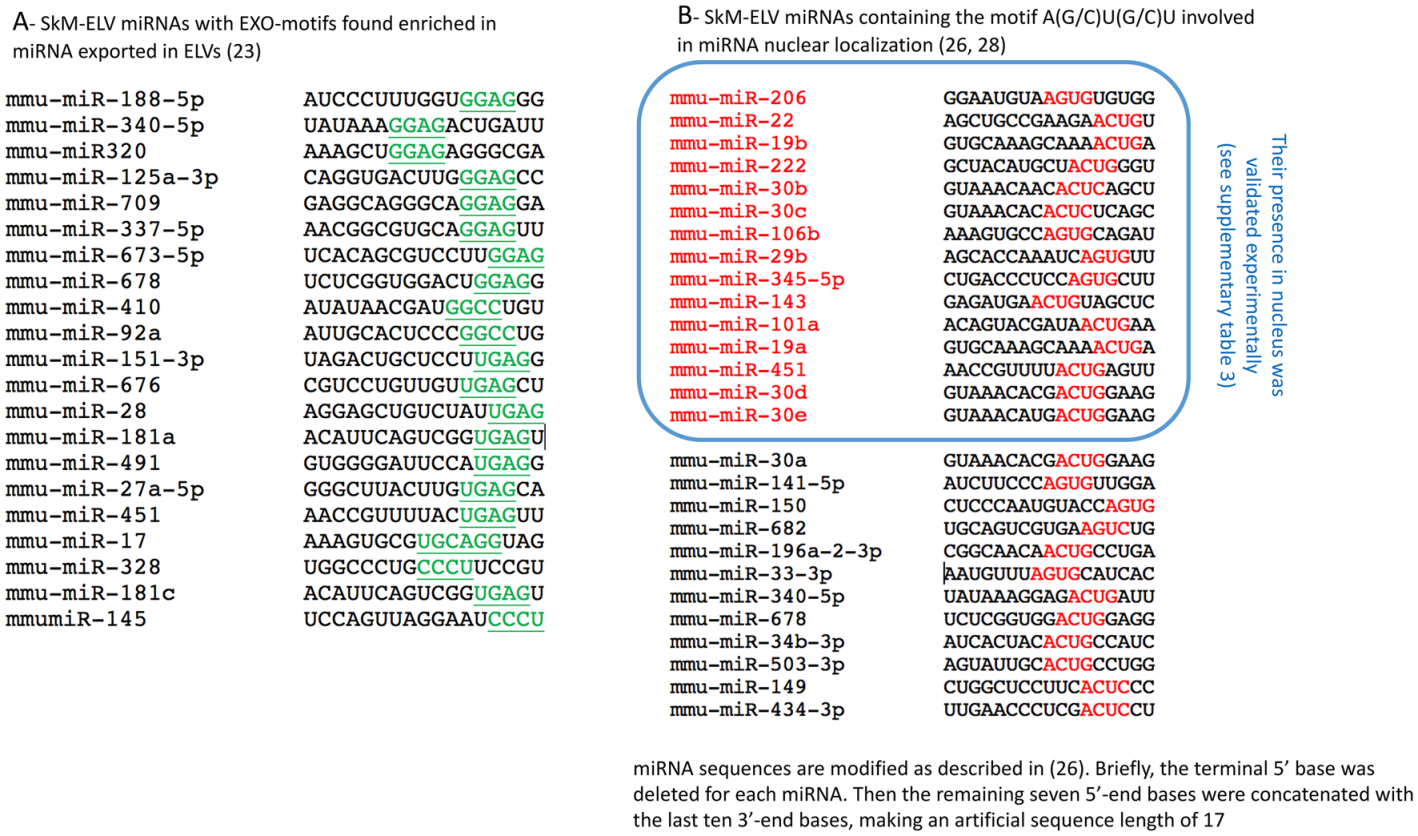
Sequence analyses of exported miRNA in SkM-ELVs. (**A**) SkM-ELV-miRNAs with EXO-motifs^23^. (**B**) Detailed procedure to identify the A(G/C)U(G/C)U sequence associated with miRNA nuclear localization. Only miRNAs contained in SkM-ELVs with this sequence are shown^26,28^. Validated miRNAs are indicated in red and the associated publications are mentioned in supplementary Table S3.

As specific lipids and proteins were differentially enriched between OB-ELVs and WT-ELVs, we wondered whether obesity also affected the concentrations of miRNAs. As for proteins and lipids, in order to take into account that SkM from OB mice released less ELVs than WT mice (Fig. 1F), total RNA was extracted from the same quantity of OB-ELVs and WT-ELVs, and the same concentration of total RNA was used for miRNA quantification (Table S3). In Fig. 4A, we have only considered the miRNAs that were consistently expressed in all animals inside the same group. Interestingly, the concentrations of the miRNAs commonly expressed between muscle and SkM-ELVs were positively correlated (Fig. 4B), demonstrating that for the majority of miRNAs, their levels in SkM-ELVs reflected their muscle concentrations, independently of the obesity context. As a previous study had also described the presence of ‘EXO motifs’ in miRNAs preferentially exported into ELVs^23^, we determined whether miRNA SkM-ELVs had ‘EXO-motifs’ in their 3’ half: 13.0% of the 165 WT-ELV miRNAs had EXO-motifs *vs* 4.5% for the not exported ones (Table S3, Fig. 5A). In addition, the mean *Ct* values of those with EXO-motifs were significantly lower in SkM-ELVs *vs* muscle (Ct = 27.3 ± 0.94 *vs* 31.06 ± 1.4, *p* value = 0.048) suggesting that the presence of these EXO-motifs would favor their incorporation into SkM-ELVs.

Among the 113 miRNAs commonly expressed between OB-ELVs and WT-ELVs, 7 were differently expressed (Table 1, Fig. 4C). This low number was in agreement with the low number of miRNAs differently expressed between OB and WT muscles (Table 1, Fig. 4D). Unexpectedly, they were not the same between ELVs or between muscles from OB *vs* WT mice (Fig. 4C *vs* Fig. 4D). This data demonstrated that obesity affected SkM-ELV miRNAs content but that this altered miRNA signature does not reflect intramuscular miRNA alterations, in OB mice.

**Table 1:** miRNAs differentially enriched between WT-ELVs *vs* OB-ELVs (n = 4 animals per group) or between quadriceps muscle of OB vs WT (n = 3).

	miRNA annotations on TLDA	miRBase 20 annotations	TLDA ID	miRBase I.D	Mean WT	SEM WT	Mean OB	SEM OB	Student *t*-test *p* values (OB vs WT)	fold changes OB/WT
Differentially expressed in SkM-ELVs	mmu-miR-434-5p		4,395,711	MIMAT0001421	32.71	0.25	ND	ND	0.000093	**− 156.22**
	mmu-miR-340-5p		4,395,369	MIMAT0004651	32.75	0.63	ND	ND	0.0015	**− 152.19**
	mmu-miR-1	mmu-miR-1a-3p	4,395,333	MIMAT0000123	22.92	0.33	25.21	0.32	0.002583041	**− 4.87**
	mmu-miR-101a	mmu-miR-101a-3p	4,395,364	MIMAT0000133	28.59	0.21	30.78	0.48	0.013	**− 4.56**
	mmu-miR-101b	mmu-miR-101b-3p	4,395,661	MIMAT0000616	28.74	0.13	30.44	0.12	0.000091	**− 3.25**
	mmu-miR-29b*	mmu-miR-29b-1-5p	4,395,627	MIMAT0004523	27.90	0.20	26.92	0.15	0.011	*1.97*
	mmu-miR-224	mmu-miR-224-5p	4,395,683	MIMAT0000671	32.49	0.44	30.55	0.20	0.015	*3.83*
Differentially expressed in quadriceps	mmu-miR-434-3p		4,395,711	MIMAT0001422	28.39	0.16	26.87	0.33	0.029234456	*2.32*
	mmu-miR-495	mmu-miR-495-3p	4,381,078	MIMAT0003456	33.41	0.15	32.19	0.18	0.007378497	*1.49*
	mmu-miR-299*	mmu-miR-299a-5p	4,373,188	MIMAT0000377	31.53	0.11	30.83	0.14	0.018629993	*1.99*
	mmu-miR-200c	mmu-miR-200c-3p	4,395,411	MIMAT0000657	31.73	0.19	32.5	0.15	0.03923314	**− 1.71**
	mmu-miR-106b	mmu-miR-106b-5p	4,373,155	MIMAT0000386	29.41	0.15	30.21	0.2	0.039560446	**− 1.58**
	mmu-miR-203	mmu-miR-203-3p	4,373,095	MIMAT0000236	29.72	0.18	30.55	0.12	0.02686222	**− 1.46**
	mmu-miR-24	mmu-miR-24-3p	4,373,072	MIMAT0000219	21.72	0.18	22.57	0.02	0.044287773	**− 1.4**
	mmu-miR-146a	mmu-miR-146a-5p	4,373,132	MIMAT0000158	26.06	0.26	27.91	0.43	0.031590388	**− 3.39**

Data are expressed as qRT-PCR C*t* values.

The C*t* is defined as the number of cycles required for the fluorescent signal to cross the threshold (*i.e.*; exceeds background level).

C*t* levels are inversely proportional to the amount of target nucleic acid in the sample (*i.e.*; the lower the Ct level the greater the amount of target nucleic acid in the sample).

In italics: miRNAs increased in OB-ELVs *vs* WT-ELVs; in bold miRNAs decreased in OB-ELVs *vs* WT-ELVs.

### OB-ELV-miRNA target genes involved in fatty acid metabolism but not located in mitochondria

As the functions of the SkM-ELV miRNAs released from muscle explants had never been studied before, we predicted their target genes and performed functional enrichment analyses to determine which pathways they affected in the recipient cells. Table 2 showed that SkM-ELV miRNAs targeted genes involved in vesicular fusions and membrane budding, and were located in endosomes and clathrin-coated vesicles. These data indicated that SkM-ELV miRNAs would control the vesicle trafficking and the dynamic of endosomes in the recipient cells (Table 2). Very interestingly, the list of SkM-ELV miRNA target genes was significantly depleted in genes located in mitochondria or having oxidoreductase activity (Table 2, Table S3). This data indicated thus that mitochondria are not targeted by SkM-ELV miRNAs in recipient cells.

**Table 2:** Gene Ontology pathways significantly enriched in genes targeted by quadriceps-released WT-ELV miRNAs.

	GO molecular component	Homo sapiens—REFLIST (20,851)	WT-ELV	WT-ELV (expected)	WT-ELV (fold Enrichment)	WT-ELV (raw P-value)	WT-ELV (FDR)
GO Functions over-represented	ER to Golgi transport vesicle membrane (GO:0012507)	17	15	5.55	2.7	6.43E−03	2.68E−02
	Dendritic spine (GO:0043197)	30	25	9.79	2.55	7.92E−04	4.85E−03
	Neuron spine (GO:0044309)	30	25	9.79	2.55	7.92E−04	4.79E−03
	Presynaptic active zone (GO:0048786)	24	20	7.83	2.55	2.51E−03	1.29E−02
	PcG protein complex (GO:0031519)	28	20	9.14	2.19	1.07E−02	4.15E−02
	Endocytic vesicle (GO:0030139)	29	20	9.47	2.11	1.21E−02	4.59E−02
	Transport vesicle membrane (GO:0030658)	61	42	19.91	2.11	3.22E−04	2.52E−03
	Postsynapse (GO:0098794)	126	85	41.13	2.07	4.91E−07	6.24E−06
	Synaptic membrane (GO:0097060)	78	52	25.46	2.04	1.33E−04	1.18E−03
	SNARE complex (GO:0031201)	39	26	12.73	2.04	5.85E−03	2.50E−02
	Synaptic vesicle (GO:0008021)	68	45	22.2	2.03	3.97E−04	2.88E−03
	Golgi apparatus subcompartment (GO:0098791)	70	46	22.85	2.01	3.27E−04	2.44E−03
	Endosome membrane (GO:0010008)	72	47	23.5	2	3.73E−04	2.75E−03
	Coated vesicle membrane (GO:0030662)	46	30	15.02	2	4.64E−03	2.14E−02
	Postsynaptic specialization (GO:0099572)	69	45	22.53	2	4.38E−04	3.01E−03
	Transport vesicle (GO:0030133)	115	75	37.54	2	6.02E−06	6.65E−05
	Cortical cytoskeleton (GO:0030863)	43	28	14.04	2	5.50E−03	2.47E−02
	Clathrin-coated vesicle (GO:0030136)	40	26	13.06	2	9.21E−03	3.66E−02
	Cytoplasmic ribonucleoprotein granule (GO:0036464)	75	48	24.48	2	4.68E−04	3.17E−03
GO Functions under-represented	Mitochondrial protein-containing complex (GO:0098798)	262	50	85.53	0.58	2.66E−04	2.28E−03
	Condensed nuclear chromosome (GO:0000794)	145	25	47.34	0.53	2.27E−03	1.55E−02
	Ribosome (GO:0005840)	225	38	73.45	0.52	6.94E−05	6.66E−04
	Myelin sheath (GO:0043209)	213	30	69.53	0.43	2.87E−06	3.15E−05
	Respiratory chain complex (GO:0098803)	80	11	26.12	0.42	4.69E−03	3.05E−02
	Mitochondrial ribosome (GO:0005761)	90	10	29.38	0.34	4.06E−04	3.30E−03
	Synaptonemal structure (GO:0099086)	97	9	31.67	0.28	3.56E−05	3.51E−04
	Respiratory chain complex I (GO:0045271)	49	4	16	0.25	2.16E−03	1.50E−02
	NADH dehydrogenase complex (GO:0030964)	49	4	16	0.25	2.16E−03	1.50E−02
	Immunoglobulin complex (GO:0019814)	192	2	62.68	0.03	3.90E−21	1.02E−19
	Immunoglobulin complex, circulating (GO:0042571)	188	1	61.37	0.02	6.31E−22	1.69E−20
	90S preribosome (GO:0030686)	28	0	9.14	< 0.01	6.03E−04	4.67E−03

Target genes predictions are from TargetScan 6.0.

Significant pathways are from PANTHER version 15.0 (PANTHER Overrepresentation Test).

Only functions with more than twofold enrichments (+ or -) are shown.

Then we determined the significant KEGG pathways commonly targeted by the altered OB-ELV miRNAs (Table 1). The most significantly affected function was predicted to be 'fatty acid metabolism' (Fig. 4E). As this function was targeted by miRNAs depleted (miR-340-5p) or less abundant in OB-ELVs *vs* WT-ELVs (*i.e*.; miR-1a-3p, miR-101a-3p, miR-101b-3p), this data suggested that fatty acid metabolism would be less repressed in recipient cells for SkM-ELVs in obese animals than in healthy animals. Considering the enriched miRNAs (*i.e*.; mmu-miR-29b-1-5p and mmu-miR-224-5p), the most significant targeted KEGG pathway in recipient cells was 'Lysine degradation (mmu00310)' (p = 4.008e-06).

### Many muscle released ELV miRNAs have a nuclear localization in recipient cells

Interestingly, we observed that miR-709 which is predominantly located in the nucleus of various cell types^24^, was highly concentrated in SkM-ELVs released either from C2C12 cells or quadriceps (Table S3). Through a literature survey investigating the subcellular distribution of miRNAs, we found that 78 SkM-ELV miRNAs had been already identified in the nucleus of various cell types^25–28^ including the muscle-specific miR-206^29^ (Table S3). Some of them had defined functions in nucleus either by targeting promoter regions (*i.e.*; miRNAs miR-320, miR-423-5p and miR-744^30^ or by cleaving non-coding antisense transcripts in human cells (i.e.; miR-671) or by regulating miRNA biogenesis at the post-transcriptional level (i.e.; miR-709)^24^. Translocation of miRNAs into the nucleus was reported to be associated with the presence of a specific motif (ASUS (S = G or C)) in their 3’ termini^26,28^. We found that 36 miRNAs out of the 165 miRNAs commonly expressed in muscle and SkM-ELVs had one of these motifs in their 3’ region (Fig. 5B). Interestingly, 'nuclear import signal receptor activity (GO:0061608)' and 'nuclear localization sequence binding (GO:0008139)' were 2 significant molecular functions of the SkM-ELV proteins (Table S2). These data indicated that in addition to miRNAs with nuclear import sequences, SkM-ELV also transfer proteins able to integrate the nucleus of the recipient cells.

Interestingly, some OB-ELV altered miRNAs, such as miR-340-5p, miR-101a-3p, miR-101b-3p and miR-224-5p had been detected in the nucleus of various cell types and miR-340-5p and miR-101a-3p have a nuclear motif in their 3’ regions (Fig. 5B) indicating that they would target the nuclear compartment in recipient cells.

### OB-ELVs modulated lipogenesis in recipient adipocytes

**Figure 6:**
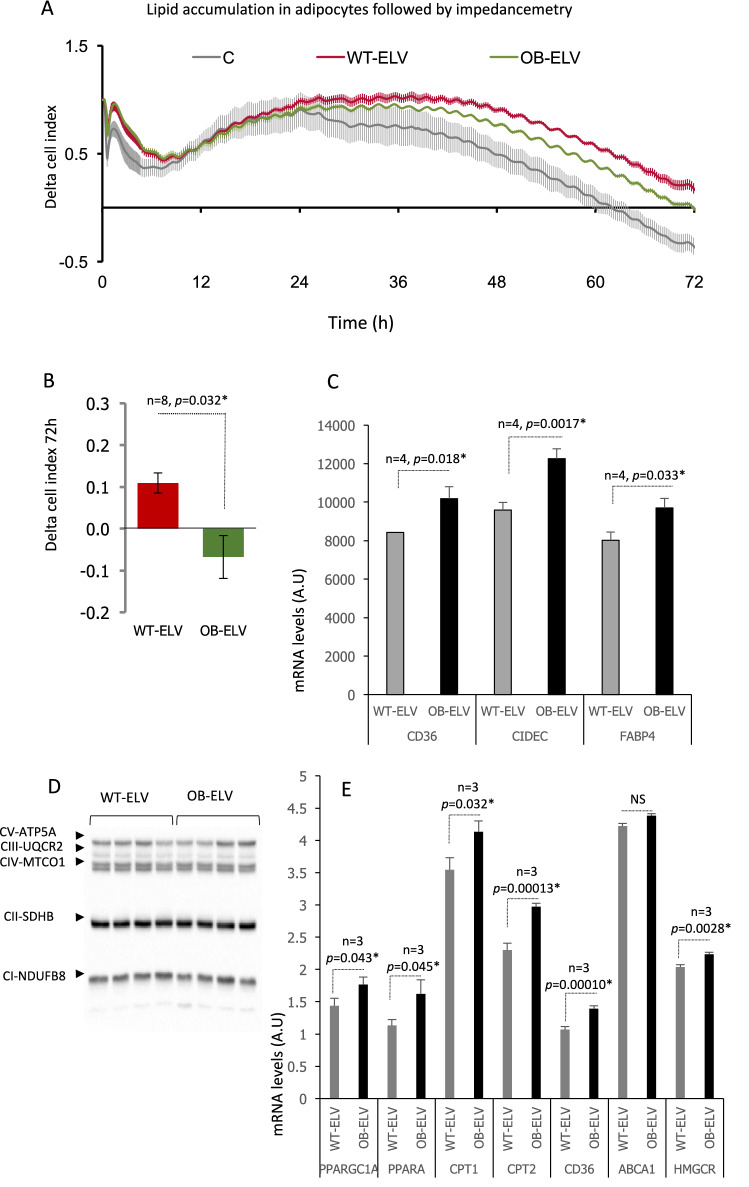
OB-ELVs modulate lipid storage in recipient cells. (**A**) 3T3-L1 cells were grown and differentiated in 96-wells E-plates and then maintained in DMEM/5% FBS vesicle-free for 2 h before treatment with OB-ELVs or WT-ELVs. Untreated adipocytes were used as control. The curves represent real-time monitoring of the cell index substracted from the cell index at T = 0 (mean delta cell index, every 5 min during 50 cycles then every 15 min) for 72 h. (**B**) Slopes at T = 72 h (mean values + /- SEM) (n = 8). (**C**) mRNA levels of genes involved in lipogenesis in differentiated adipocytes at T = 72 h post treatment with OB-ELVs or WT-ELVs. (**D**) Western-blot showing the oxphos proteins in C2C12 treated for 24 h with OB-ELVs or WT-ELVs (the full gel is shown). (**E**) mRNA levels of genes involved lipid metabolism in C2C12 treated for 24 h with OB-ELVs or WT-ELVs. For all figures, * = significant *p*-values (*p* < 0.05, student *t*-test).

Then we determined whether OB-ELVs would regulate the fate of other cell types affected during the development of obesity. As we previously demonstrated that SkM-ELVs could enter into adipocytes^11^ that are major actors in the development of obesity, differentiated 3T3-L1 adipocytes were treated with OB-ELVs and WT-ELVs. Neither adipocyte proliferation nor the induction of their differentiation, were affected (data not shown). As shown on Fig. 6A, differentiated 3T3-L1 grown in normal media displayed a strong decrease in delta cell-index as the result of a change in their morphology. We have previously demonstrated that this result is due to intracellular lipid accumulation in adipocytes^31^. Adipocytes treated with OB-ELVs or WT-ELVs displayed the same tendency as the control cells until 24 h post-treatment. After 24 h, an 'ELV effect' was detected as adipocytes accumulated less lipids in the presence of WT- or OB-ELVs than when untreated. Interestingly, lipid accumulation was higher for OB-ELVs than for WT-ELVs as shown by significantly different delta cell index (Fig. 6B), indicating that OB-ELVs had lost the capacity to retain lipid accumulation in adipocytes. This result was confirmed by quantifying mRNA levels in recipient 3T3-L1 (Fig. 6C). The levels of CD36 and FABP4, involved in lipid entrance and transport and CIDEC, promoting lipid droplet formation in adipocytes, were found higher after OB-ELVs treatment *vs* WT-ELVs, confirming higher lipid storage.

### OB-ELVs induced lipid oxidation and altered insulin-sensitivity and phenotype in recipient myotubes

Although atrophic, it has been shown that OB SkM has greater fat content (^32^ and Fig. 3B) and have increased fatty acid oxidation compared with lean mice SkM^33^. Therefore, we determined whether OB-ELVs released from OB SkM could transfer these alterations of lipid metabolism into C2C12 muscle cells. Figure 6D showed that OB-ELVs and WT-ELVs did not differentially modulate the level of mitochondrial oxphos proteins demonstrating that the number of mitochondria was not affected in recipient C2C12. Transcriptomic analyses showed that OB-ELV-treated C2C12 captured more fatty acids (increase CD36 mRNA level) which were used for mitochondrial oxidation, as indicated by the mRNA level increase of 2 major actors of energy production, PPAR alpha and PGC1 alpha (Fig. 6E). As CPT1, CPT2 and HMGCR are target genes of PGC1 alpha, their increased mRNA levels are likely due to the increased expression of PGC1 alpha (Fig. 6E). CPTs are a rate limiting step for long-chain fatty acids transport from cytosol into mitochondria for fatty acid oxidation. In contrary, ABCA1 cholesterol transporter mRNA level was not affected.

**Figure 7:**
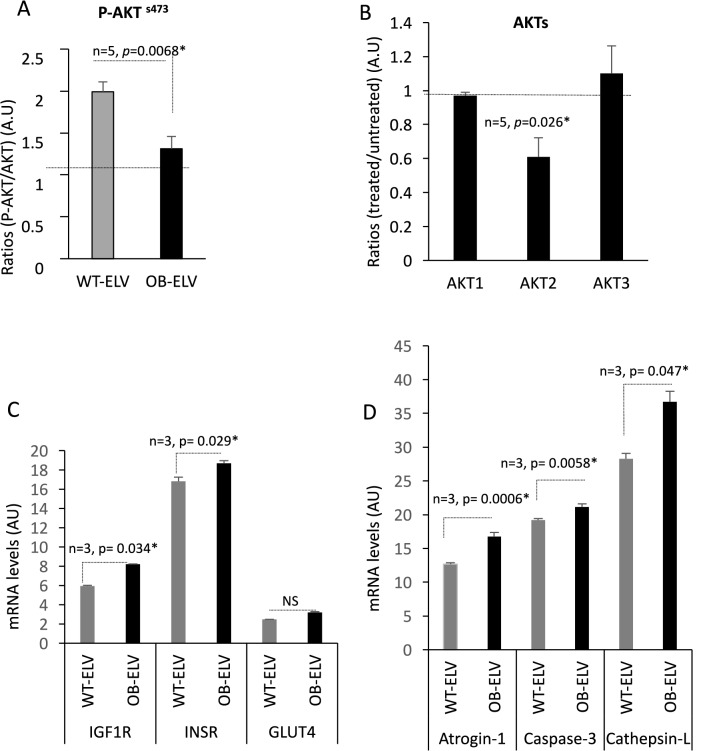
OB-ELVs induced insulin-resistance and atrophy in recipient muscle cells. (**A**) Quantification of phosphorylated AKT protein on serine serine A-473 by Western-blot in C2C12 treated with SkM-ELVs. Data are expressed in fold p-AKT/total AKT. (**B**) Quantification of AKT isoforms in C2C12 treated with SkM-ELVs by WB. Data are expressed as ratios of AKT level in treated vs untreated conditions. (**C**) mRNA levels of genes involved in insulin-stimulated glucose uptake. Data are expressed as arbitrary units. (**D**) mRNA level of genes involved in muscle atrophy. Data are expressed as arbitrary units.

As in OB mice SkM have altered insulin-sensitivity, we wondered whether ELVs released from these muscles would induce IR in recipient C2C12. Figure 7A showed that C2C12 treated with OB-ELVs had altered AKT phosphorylation in response to insulin stimulation (Fig. 7A) and showed a decrease in the quantity of AKT2 protein (Fig. 7B) when compared to cells treated with WT-ELVs. Concomitantly, mRNA levels of the receptors for IGF-1 and insulin increased, may-be as a compensatory mechanism to overcome IR in recipient C2C12 (Fig. 7C). Conversely, Glut4 mRNA was not significantly modulated (Fig. 7C). In addition, OB-ELVs treatment enhanced the production of atrogin-1 mRNA (marker of muscle atrophy^34^), of Caspase-3 and Cathepsin-L (markers of muscle protein loss^35,36^) (Fig. 7D). These data indicated that OB-ELVs induced a message of atrophy only 24 h post- treatment.

## Discussion

In this study, we have determined for the first time the consequences of obesity on the repertoire of biomolecules carried by exosome-like vesicles (ELVs) released from skeletal muscle (SkM) explants and identified the biological message associated to these modifications, *ex vi*v*o*. For this purpose, we have considered the ob/ob mice (OB) homozygous for the obese spontaneous mutation Lep^ob^, which were fed on a normal chow diet. OB mice are hyperphagic, obese, hyperinsulinemic, and hyperglycemic. As SkM is an important target for leptin, its mutation strongly affects the regulation of lipid and glucose metabolisms^37^ and induces protein degradation-associated atrophy. In addition to be insulin-resistant (IR)^38^, OB SkM have increased levels of proteins involved in fatty acid oxidation^33^, and we confirmed that OB SkMs have ectopic fat accumulation compared to lean SkMs^32^.

Considering that in OB mice the first relevant target cells of SkM-ELVs would be the muscle itself and adipocytes, we have treated these 2 cell types with OB-ELVs and WT-ELVs released from SkM explants of OB and lean mice. Very interestingly, the data showed that in these 2 recipient cells, OB-ELVs disturbed lipid metabolism, *i.e*.; they induced lipid storage in adipocytes and lipid uptake and oxidation in myotubes, *vs* WT-OB. Surprisingly, both OB-ELVs and WT-ELVs reduced lipid accumulation in adipocytes, suggesting for the first time the existence of a cross-talk between SkM and the adipose tissue (AT) through the SkM-ELV route, to prevent lipid accumulation in AT. However, in obese animals, our data demonstrated that adipocytes accumulated more lipids when treated with OB-ELVs rather than with WT-ELVs. Therefore, in obesity the message released from SkM-ELVs is modified and favors adipose tissue lipid storage and expansion. In addition to their action on lipid metabolism, our study also demonstrates that OB-ELVs would participate in the loss of muscle mass which is observed in OB animals. Indeed, OB-ELVs are able to induce IR and atrophy in recipient SkM cells, thus indicating that in OB mice, OB-ELVs act as a paracrine signal which spreads an 'atrophic signal' among muscle cells. We have previously demonstrated that insulin-resistant SkM from diet-induced-obese (DIO) mice released SkM-ELVs able to alter the expression of markers of myotube terminal differentiation (*i.e*.; decrease Myog and Myod1 expressions), but did not alter AKT phosphorylation in response to insulin^10^. In this study, OB-ELVs did not alter myotube phenotype (data not showed) but transferred IR between muscle cells. Taken together, these data demonstrated that the signal sent by OB SkM is specific to OB mice and cannot be extrapolated to all situations leading to the development of insulin-resistance, in obesity.

Based on these *ex-vivo* data, we speculated that individual or combined actions of components contained in OB-ELVs might explain their biological activities. Therefore, we focused on the miRNAs, lipids and proteins which are the major components of SkM-ELVs. Proteomic analyses demonstrated that OB-ELVs sorted a subset of proteins involved in fatty acid oxidation. In this study, we did not perform proteomic analyses of OB- and WT-SkM, but a previous proteomic study demonstrated that enzymes involved in lipid metabolism were strongly increased in OB muscles, likely as a consequence of the increased lipid availability^33^. Therefore, our data indicated that OB-ELV protein composition reflects the adaptive remodeling of lipid metabolism in muscles from OB mice. Given the fact that many of these OB-ELV proteins have catalytic activities, it would be interesting to determine whether they could be functional in the recipient cells. Indeed, recent studies have demonstrated that adipose tissue-derived ELVs from obese mice (DIO or OB mice) were also enriched in proteins involved in fatty acid oxidation^39^, and it was elegantly demonstrated that these proteins were transferred and functional into recipient cancer cells, resulting in a metabolic remodeling in favor of fatty acid oxidation which promoted cancer cell invasion^39^. Among these adipocyte-derived ELV proteins, NDUV1, HCDH, CPT2, ACADL and SCOT1, involved in energy production and lipid oxidation are also contained and enriched in OB-ELVs, suggesting also a possible role of these proteins in the action of OB-ELVs on the regulation of lipid metabolism in muscle cells and adipocytes.

Then we determined the consequence of obesity on the lipid composition of SkM-ELVs and report, for the first time, significant differences between OB and lean mice. The SkM lipidomic analysis confirmed a previous study showing that the levels of membrane lipids such as SM, PC and PE were comparable between OB-SkM and WT-SkM^40^. Therefore, it might explains that their global concentrations did not vary between OB-ELVs and WT-ELVs. Similarly, cholesterol enrichment in OB-ELVs is correlated with its increased concentration in OB muscle. It has been suggested that cholesterol in ELVs was implicated in cellular cholesterol homeostasis^41^. Thus, its increased concentration in OB-ELVs could be a way to remove its accumulation in OB muscle. However, as cholesterol is also involved in ELV release, when its intracellular concentration exceeds a threshold, the motility of late endosomes is strongly affected^42–44^ which likely explains, together with the decrease of RAB35, that OB muscle released less SkM-ELVs than lean muscle. However, the lipidomic analysis revealed that the majority of PC, SM and ceramides (except for Cer d18:1/18:0) that were altered in OB-SkM *vs* WT-SkM were not affected in OB-ELVs *vs* WT-ELVs, indicating that the sorting of these phospholipids and sphingomyelin in SkM-ELVs is strongly regulated to stay at a constant level whatever the pathological context, or that these lipids are not involved in SkM-ELVs biogenesis. For few lipids, there was a discrepancy between their variations in muscle and in SkM-ELVs. Indeed, OB-SkM accumulated specific isomers of PC (34:4 and 40:3), depleted in OB-SkM, and SM (d18:1/18:1), not affected in OB-SkM. SM and cholesterol confer rigidity to ELV membranes, and modifications of their concentrations in OB-ELVs could affect OB-ELVs fate and their incorporation into recipient cells. In line with this hypothesis, a recent study has demonstrated that blood-derived ELVs were preferentially internalized by circulating leucocytes when they originated from patients suffering from diabetes when compared to those from normoglycemic patients^45^.

Considering that SkM-ELVs are from endosomal origin and are formed inside MBVs, the OB-ELV lipidomic data also indicate that obesity affects MVB lipid composition. How does it modify the physico-chemical properties of the MVB bilayers, for curvature, lipid, protein and miRNA incorporation, electrostatics^46^ and signaling pathway involving AKT^5^ is currently unknown and would deserve further studies.

Then we focused on the miRNA population contained in SkM-ELVs and determined how it was affected by obesity. We have previously demonstrated that SkM-ELV miRNAs can enter into recipient cells and regulate gene expression^11,47^, thus it was beyond the scope of this study to reproduce these data. Here, we focused on the subcellular compartments that are predicted to be targeted by SkM-ELV miRNAs in recipient cells, which had never been taken into account before. As many miRNA targets are predicted by bioinformatics, some are false positive and need to be individually validated. In order to overcome this problem, we have chosen to consider all miRNA targets at the same time, and to focus on the cellular pathways affected by a significant number of predicted genes. This analysis demonstrated that SkM-ELV miRNAs targeted preferentially pathways involved in the traffic of vesicles between different compartments (trans-golgi network, endosome, exocytosis). Surprisingly, we found an unexpected close relationship with the nucleus, as many released miRNAs had a nuclear addressing sequence^26,28^. First, this result suggested that the nucleus is able to send these miRNAs into MVBs, in which they will be packed inside SkM-ELVs and released. Previous data have described MVB-like structures containing numerous vesicles in the nucleus^48^ and nuclear membrane budding^49^, suggesting that that components from the nucleus can traffic to the late endosomes. In favor of this hypothesis, proteomic data contain 33 proteins from the nuclear envelope in SkM-ELVs. Secondly, this result also indicated that SkM-ELV recipient cells would receive miRNAs that are able to translocate into their nuclei to exert transcriptional or epigenetic controls^30^, in addition to the control of protein concentrations in the cytoplasm. This has never been suggested before, and would deserve further analyses. However, at the present time, sensitive tools are lacking for tracking SkM-ELV-miRNAs once they are incorporated into recipient cells. Finally, we found that mitochondria are not targeted by SkM-ELV miRNAs. This result is in agreement with the results from an early study on miRNAs demonstrating that oxidative phosphorylation was a function significantly under-represented among the miRNA targets^50^. In this study, we demonstrated that it is also the case for miRNAs exported into SkM-ELVs.

As the miRNA function is to repress protein translation^51^, it implies that if miRNAs are depleted in SkM-ELVs, the expression of their targeted mRNA will be less repressed in the recipent cells. Therefore, as depleted OB-ELV miRNAs targeted genes involved in lipid metabolism, this function would be less repressed by SkM-ELVs in obese mice. This results could explain, in part, that lipid oxidation in muscle cells treated with OB-ELVs is higher than when treated with WT-ELVs. In addition, OB-ELV are also enriched in miRNAs predicted to target mRNAs involved in the regulation of 'Lysine degradation'. This pathway is known to prevent muscle wasting, by suppressing myofibrillar protein degradation through inhibition of the autophagic-lysosomal system^52^. Therefore, through the modulation of its miRNA population released in SkM-ELVs, SkM can both increase lipid metabolism and concomittantly alter mice muscle mass though the release of 'atrophic miRNAs' in OB-ELVs.

## Conclusions

Taken together, these data demonstrated that SkM-ELVs have a very specific composition which matches only partially with the compositions of the ELV-releasing muscle in terms of lipids and miRNAs. Our data suggest for the first time that SkM-ELVs exert their actions at different levels in the recipient cells (transcription, protein levels) and, more importantly, concomitantly into different organelles, sheding new light on the mechanisms of action of SkM-ELVs in recipient cells. In OB mice, our data indicate that OB-ELVs exert a paracrine signal inside the muscle, able to transmit and maintain IR and atrophy among muscle cells. In addition, we highlight an unexpected function of OB-ELVs in the control of lipids accumulation in adipocytes which might be very important for the control of the muscle mass/adipose tissue ratio in the body. Indeed, it is known that there is an inverse correlation between the muscle mass/ strength and the quantity of adipose tissue; i.e.; increased muscle mass in Myostatin knockout mice is associated with reduced adipose tissue^53^. On the other hand, massive development of the adipose tissue in obese subjects is known to induce sarcopenia, and to decrease muscle mass and function^54^. Until now, this relationship was only explained with the release of specific myokines and adipokines which could modulate the metabolism of both tissues^55^. But recently a possible cross-talk between adipocytes and muscle cells has been described, through adipocyte-derived ELVs; *i.e*., miR-27a released from adipocytes of DIO obese mice was associated with TAG accumulation and IR in recipient muscle cells^56^. In this study, we demonstrate that SkM is also able to modulate the adipose tissue function through the release of SkM-ELVs. Therefore, like myokines and adipokines, the exchange of ELVs between adipose tissues and SkM represents a new way of communication that has to be taken into account to develop new therapeutic strategies.

## Supplementary Information

Supplementary Information 1.

Supplementary Information 2.

Supplementary Information 3.

## Abbreviations

ELV: Exosome-like vesicles
MVB: Multivesicular bodies
ILV: Intraluminal vesicles
GAST: Gastrocnemius
QUAD: Quadriceps
PGC1 α (PPARGC1A): Peroxisome proliferator-activated receptor gamma coactivator 1-alpha
INSR: Insulin receptor
PPAR gamma: Peroxisome proliferator-activated receptor gamma
IGF1R: Insulin-like growth factor 1 receptor
ABCA1: Phospholipid-transporting ATPase ABCA1
AKT: RAC-alpha serine/threonine-protein kinase
CD36: Cluster of differentiation 36
FABP4: Fatty acid-binding protein 4
CIDEC: Cell death activator CIDE-3
HMGCR: 3-Hydroxy-3-methylglutaryl-coenzyme A reductase
CD81: Cluster of Differentiation 81
ALIX: Programmed cell death 6-interacting protein
TSG101: Tumor susceptibility 101
CD63: CD63 antigen
VPS4: Vacuolar protein sorting-associated protein 4A
GLUT4: Solute carrier family 2, facilitated glucose transporter member 4
