# Supplementary material for: Profiling of ob/ob mice skeletal muscle exosome-like vesicles demonstrates combined action of miRNAs, proteins and lipids to modulate lipid homeostasis in recipient cells
Source: Sci Rep. 2021 Nov 3;11:21626. doi: 10.1038/s41598-021-00983-3 (PMC8566600; doi:10.1038/s41598-021-00983-3)

SUPLEMENTARY MATERIALS

Profiling of ob/ob mice skeletal muscle exosome-like vesicles demonstrates combined action of miRNAs, proteins and lipids to modulate lipid homeostasis

Audrey JALABERT[(1)], Laura REININGER[(2)], Emmanuelle BERGER[(1,3)], Yohann COUTE[(4)], Emmanuelle MEUGNIER[(1)], Alexis FORTERRE[(1,2)], Elizabeth ERRAZURIZ-CERDA[(5)], Alain GELOEN[(1,3)], Myriam AOUADI[(5)], Karim BOUZAKRI[(2)], Jennifer RIEUSSET[(1)] and Sophie ROME[(1,7)#]

[(1)] CarMeN Laboratory (INSERM 1060, INRAE 1397, INSA), University of Lyon, Lyon-Sud Faculty of Medicine, Oullins, FRANCE
[(2)] UMR DIATHEC, EA 7294, Centre Européen d'Etude du Diabète, Université de Strasbourg, Strasbourg, FRANCE
[(3)] UMR Ecologie Microbienne Lyon (LEM), CNRS 5557, INRAE 1418, University of Lyon, VetAgro Sup, Villeurbanne, FRANCE
[(4)] Univ. Grenoble-Alpes, Inserm, CEA, UMR BioSanté U1292, CNRS CEA FR2048, Grenoble, FRANCE
[(5)] CIQLE, Claude Bernard Lyon 1 University, Lyon, FRANCE
[(6)] Centre for Infectious Medicine, Department of Medicine, Karolinska Institutet, Huddinge, SWEDEN
[(7)] Institut of Functional Genomic, ENS-Lyon, University of Lyon, CNRS 5242, INRAE Lyon, France

#Corresponding author: Sophie Rome, srome@univ-lyon1.fr

LEGENDS:

Supplementary Figure S1: **Metabolic parameters of ob/ob mice**. N=10 mice/group, **A)** Body weight (g) at sacrifice. **B)** Fasted glycemia. **C)** Liver weight at sacrifice. **D)** WB showing insulin-induced AKT phosphorylation on serine A-473 and total AKT expression, measured in gastrocnemius explants incubated *ex-vivo* with or without (control) 10^{-7}M insulin for 15mn. Actin is shown as a control of protein content in each line. Each protein was detected on an separated gel. **E)** gastrocnemius and **F)** quadriceps weight at sacrifice. *= p<0.05 (Student *t*-test, ob/ob *vs* wt mice).

Supplementary Figure S2: Full-length gels used for Figure S1, Figure 1 and Figure 6.

Table S1: list of antibodies used for WB or electron microscopy, and primers used for qRT-PCR.
Table S2: List of proteins identified in SkM-ELVs released from quadriceps of ob/ob mice or C57Black6 mice used as controls, and list of proteins differentially expressed in OB-ELVs vs WT-ELVs.
Table S3: List of SkM-ELV-miRNAs released from mouse OB-Quad and WT-Quad, and list of proteins expressed in nucleus.

Supplementary Figure S1

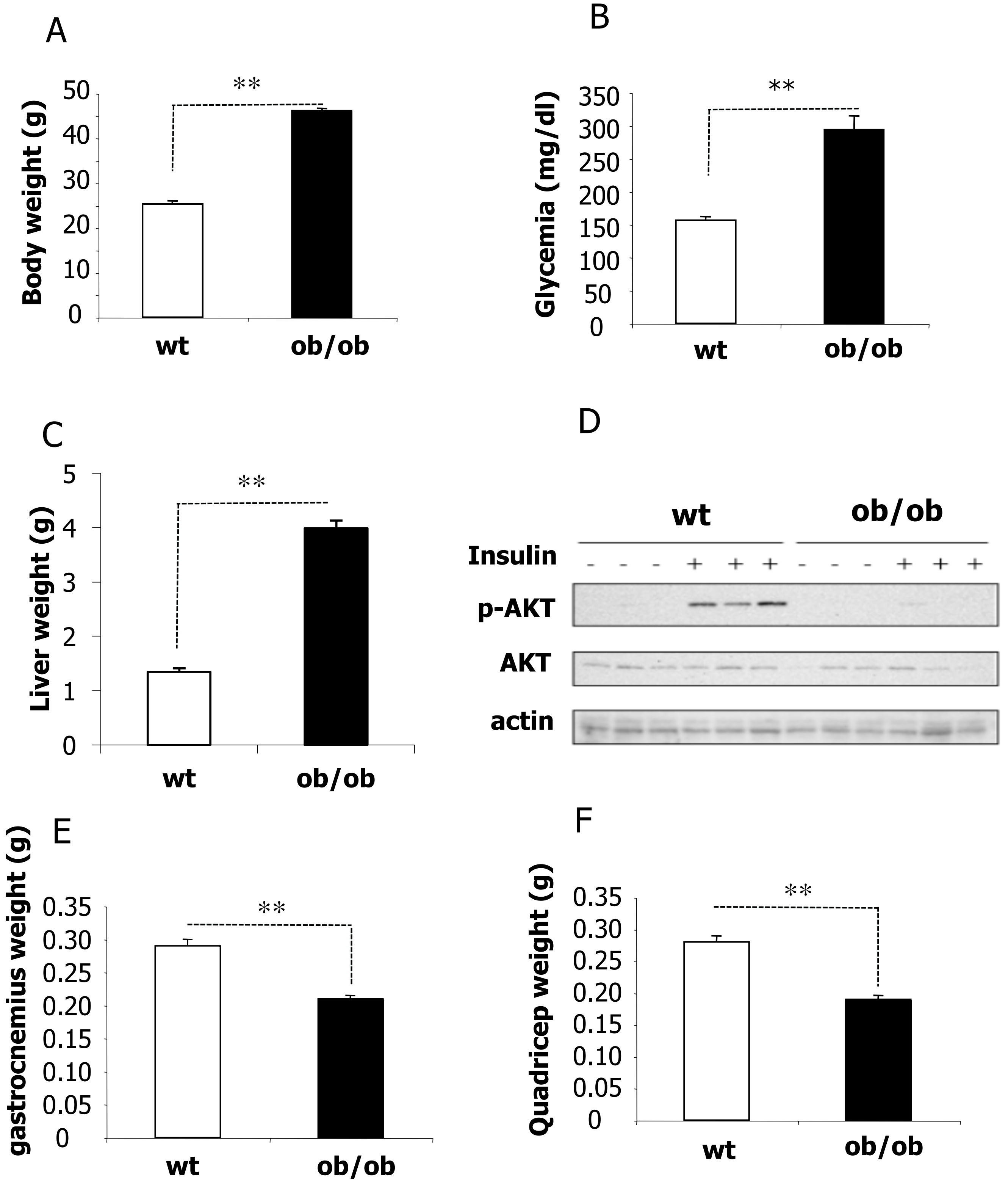

Supplementary Figure S2

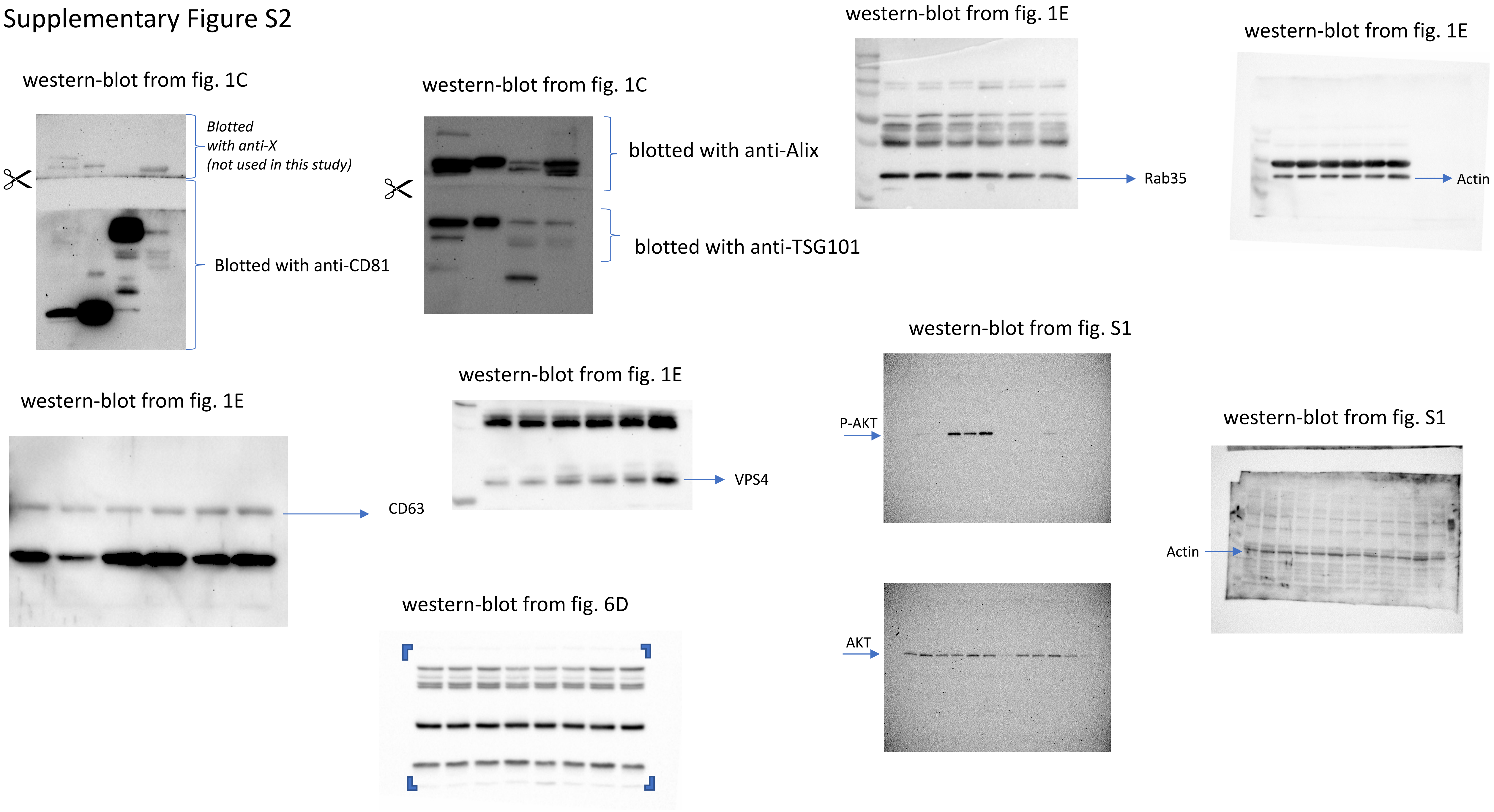

Supplement: Supplementary file 1 — Supplementary Information 1. [file 41598_2021_983_MOESM1_ESM.pdf]
